# Biofilm Formation of *Pseudomonas aeruginosa* in Cystic Fibrosis: Mechanisms of Persistence, Adaptation, and Pathogenesis

**DOI:** 10.3390/microorganisms13071527

**Published:** 2025-06-30

**Authors:** Dayana Borisova, Tsvetelina Paunova-Krasteva, Tanya Strateva, Stoyanka Stoitsova

**Affiliations:** 1 Stephan Angeloff Institute of Microbiology, Bulgarian Academy of Sciences, Acad. G. Bonchev Str., Bl. 25, 1113 Sofia, Bulgaria; daqanara@abv.bg (D.B.); stoitsova_microbiobas@yahoo.com (S.S.); 2 Department of Medical Microbiology “Corr. Mem. Prof. Ivan Mitov, MD, DMSc”, Faculty of Medicine, Medical University of Sofia, 2 Zdrave Str., 1431 Sofia, Bulgaria; dr.strateva@abv.bg

**Keywords:** cystic fibrosis, *Pseudomonas aeruginosa*, biofilms, virulence factors, antibiotics, persistence

## Abstract

Cystic fibrosis (CF) is a life-limiting autosomal recessive disorder affecting a large number of individuals in Europe. The disease arises from mutations in the CFTR gene encoding the cystic fibrosis transmembrane conductance regulator, a chloride ion channel crucial for maintaining epithelial ion and fluid homeostasis. Dysfunctional CFTR disrupts mucociliary clearance, particularly in the respiratory tract, resulting in persistent bacterial colonization, chronic inflammation, and progressive pulmonary damage—ultimately leading to respiratory failure, the principal cause of mortality in CF patients. Early diagnosis and advances in therapy have substantially improved both survival and quality of life. A hallmark of CF pathology is the establishment of polymicrobial infections within the thickened airway mucus. *Pseudomonas aeruginosa* is the dominant pathogen in chronic CF lung infections and demonstrates a remarkable capacity for adaptation via biofilm formation, metabolic reprogramming, and immune evasion. Biofilms confer increased tolerance to antimicrobial agents and facilitate long-term persistence in hypoxic, nutrient-limited microenvironments. *P. aeruginosa* exhibits a wide range of virulence factors, including exotoxins (e.g., ExoU, ExoS), pigments (pyoverdine, pyochelin), and motility structures (flagella and pili), which contribute to tissue invasion, immune modulation, and host damage. During chronic colonization, *P. aeruginosa* undergoes significant genotypic and phenotypic changes, such as mucoid conversion, downregulation of acute virulence pathways, and emergence of hypermutator phenotypes that facilitate rapid adaptation. Persistent cells, a specialized subpopulation characterized by metabolic dormancy and antibiotic tolerance, further complicate eradication efforts. The dynamic interplay between host environment and microbial evolution underlies the heterogeneity of CF lung infections and presents significant challenges for treatment. Elucidating the molecular mechanisms driving persistence, hypermutability, and biofilm resilience is critical for the development of effective therapeutic strategies targeting chronic *P. aeruginosa* infections in CF.

## 1. Introduction

CF is an inherited disease. About 35,000 children and adults in Europe suffer from CF. Annually, approximately 2000 newborns of European origin are affected by the disease [1]. Patients who are not diagnosed in time have a high risk of death at an early age. The therapy and treatment of CF patients have advanced significantly in the last decade, allowing people with this disease to have a longer life and better quality of life. One of the key factors in achieving this is early diagnosis. The disease is the result of changes in the CFTR (cystic fibrosis transmembrane conductance regulator) protein, encoded by a mutated gene [2]. This protein functions as an ion channel, regulating the transport of chloride and other ions across cell membranes. Mutations lead to an imbalance in ion transport across epithelial cells, especially in the respiratory and digestive systems. In the airways, this leads to breathing difficulties and blockage of the pulmonary alveoli, increasing the risk of infection. Persistent infections and inflammation lead to chronic lung damage and respiratory failure, which is the main cause of death in people with CF [2].

CF affects multiple organs and systems in the patient’s body. Some of the symptoms are elevated blood sugar, liver changes and infertility in men with CF, and others. The sweat chloride test is the standard way to diagnose CF [3,4]. The accumulated mucus creates a specific environment that predisposes the patient to the development of infections. In CF patients, the reduced pH of the medium, the accumulated mucus on the airway surface, and the reduced secretion of chloride ions interfere with the innate immunity’s ability to eliminate bacteria. The obstruction of the airways of the respiratory system creates conditions for colonization with opportunistic pathogens. This initiates a series of infections, inflammation, and tissue remodeling. If left untreated, this leads to a continual decline in lung function and ultimately to the premature death of patients due to respiratory failure [5]. The etiological progression of bacterial infections in cystic fibrosis patients begins with infection with *Haemophilus influenzae* in infancy and with *Staphylococcus aureus* at a slightly later stage. Typically, *P. aeruginosa* is identified as the leading etiologic agent of bronchopulmonary infections in adolescence and adulthood, although occasionally, the pathogen can be isolated in children before the age of 1 year [6]. After the initial colonization of *P. aeruginosa*, the infection progresses to a stage of sporadic or intermittent colonization in which positive and negative cultures alternate [7]. The evolution of the disease leads to the development of chronic colonization first and subsequently of chronic-recurrent infection, in which mostly mucoid strains of *P. aeruginosa* are isolated, often forming a structured community of microorganisms, a biofilm, encased in a polysaccharide matrix that they produce themselves [8]. This matrix protects the cells in the biofilm and makes them much more resistant to external agents, including antibiotics [9]. Besides the well-studied CF pathogens *P. aeruginosa*, *S. aureus*, *H. influenzae*, and *Burkholderia cenocepacia*, the lung microbiome may also contain other pathogenic species, such as *Achromobacter xylosoxidans*, *Streptococcus milleri*, *Ralstonia* spp., *Pandorea* spp., *Stenotrophomonas maltophilia*, and *Mycobacterium* spp. In addition, oral bacteria also find their way into the airways of CF patients and become part of the bacterial community, with *Rothia mucilaginosa*, *Gemella haemolysans*, and several anaerobic species being the most commonly isolated. Anaerobes, such as *Prevotella* spp., *Veillonella* spp., and *Fusobacterium* spp., are increasingly recognized as more than innocently present, given evidence that they can persist in the lungs of CF patients [10].

The complex bacterial community of the airways develops throughout a patient’s lifetime and is influenced by external factors. In older CF patients, the sputum microbiome typically becomes less diverse, which is associated with impaired lung function and consequently poorer outcomes [11]. It has been hypothesized that bacterial species present in the lung microbiome of CF patients may interact with each other when in close proximity [12].

## 2. *P. aeruginosa* Biofilms—Structure and Functions of the Extracellular Matrix

**Figure 1:**
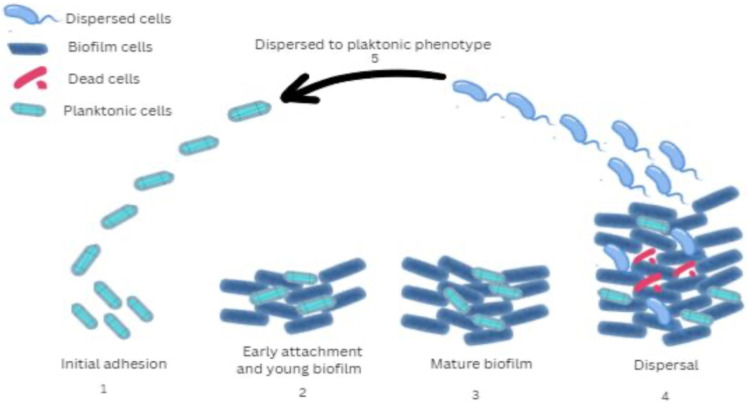
Stages of biofilm development by *P. aeruginosa*: 1 (Initial adhesion)—Bacterial surface attachment and production of extracellular polymeric substances; 2 (Early attachment and young biofilm)—Stable surface attachment, bacterial multiplication, formation of microcolonies, and intercellular interactions; 3 (Mature biofilm)—Development of a mushroom-shaped biofilm structure associated with increased resistance to antibiotic therapy; 4 (Dispersal)—Structural cavity formation within the biofilm matrix facilitates the detachment and release of individual bacterial cells; 5 (Dispersed to planktonic phenotype)—Increased attachment and enhanced virulence. The gene expression of microorganisms in biofilms is similar in many of its characteristics to that of bacteria in the stationary phase, characterized by reduced metabolic activity. A behavioral gradient depending on cell position is observed within the biofilm. The deep layers of the biofilm are often anaerobic, and these cells activate genes that are adapted for life without oxygen. In contrast, the superficial layers are rich in oxygen, providing conditions for faster growth and intense metabolic activity [21]. The presence in the biofilm of different subpopulations of bacteria in terms of rate and characteristics of metabolic processes is a prerequisite for increased tolerance to antibacterial and disinfectant agents, as well as to immune responses of the microorganism. Such tolerance occurs in biofilm cells irrespective of antibiotic susceptibility or resistance of the strain. Tolerance is reflected in significantly higher antibiotic minimal inhibitory values for the biofilm compared to free bacteria of the same strain cultured in liquid medium. This difference can be as much as 100–1000-fold [17].

Biofilms are bacterial communities that adhere to organic or inorganic surfaces. They are surrounded by a matrix that contains exopolysaccharides, DNA, proteins, and small signaling molecules [13]. Because of their structure, biofilms have the ability to respond to various external factors, such as the concentration of cells of the same or different bacterial species, the availability and quantity of nutrients, oxygen, energy sources, and other factors. Biofilm formation is considered an adaptive survival strategy under adverse environmental conditions [14]. The biofilm of *P. aeruginosa* (Figure 1) has a complex spatial structure that consists of multiple microcolonies. The mushroom-like structures are particularly interesting because they consist of a “stalk” and a “cap”. During biofilm growth, bacteria orient and reorganize themselves through various mechanisms, such as chemotaxis, motility, and quorum sensing, etc. The role of flagella is of particular importance as they are key to bacterial motility. They allow bacteria to navigate toward optimal colonization sites and initiate surface attachment [15].

Bacteria-forming biofilms show greater resistance to various xenobiotics, biocides, etc. One of the key characteristics of biofilms is the ability to tolerate significantly greater concentrations of antimicrobials than in free-living (planktonic) form, a phenomenon known as antimicrobial tolerance [4,16]. *P. aeruginosa* in biofilms localized in the lungs of CF patients are also resistant to antimicrobial treatment due to their localization in characteristic microenvironments with low oxygen levels, the presence of DNA and actin, which are products of cell necrosis of neutrophil phagocytes, etc. [17]. Bacteria with low metabolic activity and slower growth are localized in areas of the biofilm with limited access to nutrients. These almost dormant cells, due to their suppressed metabolism, reduced cell wall synthesis, etc., are accordingly characterized by increased tolerance to antimicrobials, even when the strain is sensitive to an antibiotic [18]. Furthermore, the spatial proximity of bacterial cells increases the possibility of horizontal gene transfer, including antibiotic resistance genes [19].

Bacterial cells in biofilms and those in liquid culture have different characteristics and behaviors. This leads to specificity in the diagnosis and treatment of biofilm-associated infections. While biofilm formation can be detected using a light microscope, pinpointing the specific bacterial species within it is challenging. Studies related to bacterial gene expression of bacteria in biofilms have revealed significant changes in their functionality and biochemical processes [20].

Biofilm-forming bacteria show resistance through a combination of traditional resistance mechanisms and the unique characteristics of the biofilm itself. These mechanisms may include enhanced production of enzymes such as chromosomal beta-lactamase, which degrades beta-lactam-type antibiotics, or compounds such as lipopolysaccharide, which contributes to resistance against different types of antibiotics [22]. Biofilm changes the way bacteria integrate into the host. Until recently, biofilms were assumed to be primarily associated with infections characterized by chronic inflammation, tissue damage, and serious pathological changes [17]. There are numerous studies highlighting the fact that in CF, the bacterium *P. aeruginosa* is often found in biofilm or biofilm-like aggregates in the lungs. This has been confirmed by microscopic analyses of patient samples, as well as by data on antibiotic resistance and changes in intercellular communication between bacteria, more specifically in the equilibrium between *P. aeruginosa* 3-3′-oxo-dodecanoyl homoserine lactone and butanoyl homoserine lactone signaling molecules [18]. *P. aeruginosa* forms an extracellular matrix that contains mainly extracellular polysaccharides. Polysaccharides make it difficult for antimicrobials to reach the bacterial cells, which is why CF patients often suffer from long-term lung infections. The interaction between cations, which are present in elevated concentrations in the lungs in CF, and anions strengthens biofilm structure and enhances bacterial virulence [23]. Exopolysaccharides provide physical protection from antibiotics and create distinct microenvironments with associated metabolic and phenotypic heterogeneity [24]. In 1964, Alfred Linker [25] discovered that in mucoid biofilms, *P. aeruginosa* produces the polysaccharide alginate, which contains β-D-mannuronic acid units and α-L-guluronic acid units [25]. Later, the same investigator studied polysaccharides from different strains of *P. aeruginosa* isolated from CF and found that this polysaccharide had a large molecular mass and contained predominantly β-D-mannuronic acid. Alginate has a pronounced negative charge because of two carboxylate groups on each monomer. This charge is retained in the lung environment of people with CF, where the pH value ranges between 6.85 and 7.65. The medium becomes more acidic during exacerbation of infection in CF [26]. The “screen effect” that alginate performs due to its ability to bind aminoglycosides creates biofilms with different layers. While superficial areas can become saturated with antibiotics, antibiotics do not effectively penetrate into more internal parts. Fixation of cationic drugs does not compete with Ca^2+^ sites binding to alginate [26]. The cyclic di-GMP molecule stimulates transcription of alginate production genes in *P. aeruginosa*. Furthermore, it plays a role in the regulation of alginate synthesis at the posttranslational level. Specifically, the Alg44 protein, part of the complex responsible for alginate synthesis, activates alginate polymerization upon c-di-GMP binding. Overproduction of alginate enhances the ability of *P. aeruginosa* to cause and maintain chronic infections by increasing its resistance to antibiotics. In the lungs of CF patients, *P. aeruginosa* strains that overproduce alginate are often associated with long-lasting infections. Alginate acts as a major component of the biofilm, protecting the bacteria from the body’s immune defenses and antibiotic treatment [26]. The importance of alginate and its role in resistance to infection suggests that the conditions in the CF lung are critical for the mucoid phenotype of *P. aeruginosa*. However, even without the presence of alginate, the bacterium can still form biofilms, albeit with a reduced ability to adhere to surfaces. In addition to alginate, *P. aeruginosa* produces two other exopolysaccharides, Pel and Psl, which are also involved in biofilm structure. The synthesis of these exopolysaccharides is regulated by cyclic di-GMP at different stages. For example, Psl synthesis is regulated at the transcriptional level, whereas Pel synthesis depends on c-di-GMP at both the transcriptional and posttranslational levels [27].

The function of individual polysaccharides has been elucidated through an antibiotic tolerance assay on a selection of genetically modified *P. aeruginosa* strains, which were able to overproduce one, two, or all three exopolysaccharides, alginate, Pel, and Psl [27]. The biofilms of nonmucoid forms of *P. aeruginosa*, which mainly depend on these two polysaccharides, decrease over time and are not as stable as those of mucoid forms. Furthermore, infections in the lungs caused by these forms are usually amenable to treatment, whereas infections from the alginate-rich mucoid forms of *P. aeruginosa* are more resistant to treatment [26]. Nonmucoid variants of *P. aeruginosa* are usually the first to colonize the lungs of people with CF. They synthesize the exopolysaccharides Pel and Psl. During the infection process, these nonmucoid strains may undergo genetic changes that convert them to a mucoid phenotype by starting to produce alginate. Pel, Psl, and alginate are key to the bacteria’s resistance to antimicrobial agents, as they help block the penetration of antibiotics into the biofilm formed by *P. aeruginosa*. Separately, Psl facilitates the integration of *P. aeruginosa* into microbial communities forming polymicrobial biofilms [28].

The exopolysaccharide Psl plays an important role in biofilm formation and stability, as well as in bacterial resistance to antibiotics. This fact may account for the difficulty in treating *P. aeruginosa* infections in the respiratory system of CF patients [29]. Psl is a neutral polysaccharide and is perceived as a key factor in biofilm structure and protection from immune responses. While mucoid strains produce less Psl compared to nonmucoid strains, the Psl produced has an active function by mediating adhesion to human airway cells and inducing epithelial cell apoptosis. The presence of Psl in mucoid strains induces a proinflammatory response in the lungs, leading to decreased colonization [28]. In the process of infection, *P. aeruginosa* changes to adapt to the challenges of the infection environment. A further adaptation is the transition to the small colony phenotype. This phenotype is characterized by enhanced production of the exopolysaccharides Pel and Psl, which are key for biofilm formation and stability, as well as for protection by the immune system and various antibacterial agents. Extracellular DNA (eDNA) is crucial for bacterial aggregation and for their interaction with polymorphonuclear leukocytes during the immune response. These observations highlight that in in vivo infections associated with *P. aeruginosa* biofilms, much of the eDNA is localized outside the biofilms themselves [29].

## 3. Virulence Factors in *P. aeruginosa*

Metabolic adaptability, antibiotic resistance, biofilm establishment, and the generation of multiple virulence factors make *P. aeruginosa* a particularly dangerous pathogen [1,30]. *P. aeruginosa* possesses a multiplicity of virulence factors that can be cell-associated or extracellular [31,32]. The cell-associated virulence factors comprise lipopolysaccharide (LPS), outer membrane proteins and lipoproteins, flagella, and type IV pili. Extracellular virulence factors include both proteins and secondary metabolites, e.g., siderophores, rhamnolipids, and pyocyanin [32]. The alkaline protease is secreted by *P. aeruginosa* via a type I secretion mechanism. The type II system exports various enzymes and toxins into the environment, including the elastases LasA and LasB, the exotoxin A lipase phospholipase C, and protease IV.

Exotoxin A is a mono-ADP-ribosyltransferase and is an extremely potent toxin. By ADP-ribosylation of proteins in eukaryotic cells, it disrupts their protein synthesis. The toxin is released as a protoenzyme that is also toxic to cultured cells and living organisms [33,34]. *P. aeruginosa* produces pigments such as pyoverdine, pyochelin, pyocyanin, and pyomelanin. When there is insufficient iron in the medium, the bacterium increases the production of pyocyanin and pyoverdine, as they act as siderophores. Pyoverdine is a fluorescent pigment that helps the bacterium uptake iron, while pyocyanin is a blue pigment that increases the aggressiveness of the bacterium by interfering with host cells [35,36]. Pyochelin is a siderophore with less affinity for iron but can induce inflammation. Pyomelanin is a brown pigment associated with chronic infections [37,38].

### 3.1. Lipopolysaccharide

LPS, which is a part of the outer membrane of the cell wall of most Gram-negative bacteria, is a key virulence factor in *P. aeruginosa*. Lipopolysaccharide not only protects the bacterium from external factors, it also serves as a target for the host immune system [39]. *P. aeruginosa* can simultaneously produce two O-antigens: the general polysaccharide antigen and the O-specific antigen. The common polysaccharide antigen is found in multiple strains and has a structure consisting of repeating units →3)D-Rha(α1→3)D-Rha(α1→2)D-Rha(α1→. O-specific antigens have a more diverse structure, repeating in groups of strains with a common serotype, and are therefore the determinant used in serotyping this bacterial species [39]. Structural changes of lipid A in *P. aeruginosa* from the respiratory tract of CF patients include three possible alterations. The first change, which is observed in the majority of CF isolates, is the O-linking of palmitate to the 3-OH group at the 3′ position of the glucosamine monosaccharide. In other CF isolates, a non-stoichiometric addition of aminoarabinose to the terminal phosphate residues of lipid A is observed. The third alteration, heptahydrated-acetylated lipid A, is specific only to CF isolates of *P. aeruginosa* [40]. The O-specific antigen plays a key role in the formation of various aggregation forms of *P. aeruginosa*, which is attributed to changes in the hydrophobic properties of the cell envelope. During long-term infections, e.g., in CF, the interaction between bacterial surface characteristics and environmental polymers may influence the structural properties and formation of bacterial aggregates [41]. Lipopolysaccharide is actively involved in the interaction of the bacterium with its environment. Both hydrophobic and polar properties of lipopolysaccharide reduce membrane permeability. The lipid layer prevents the penetration of polar molecules, while the polar portions of lipopolysaccharide block lipophilic compounds [39]. This lipopolysaccharide functions as a physical barrier, reducing the penetration of antimicrobials into the cell [42].

**Figure 2:**
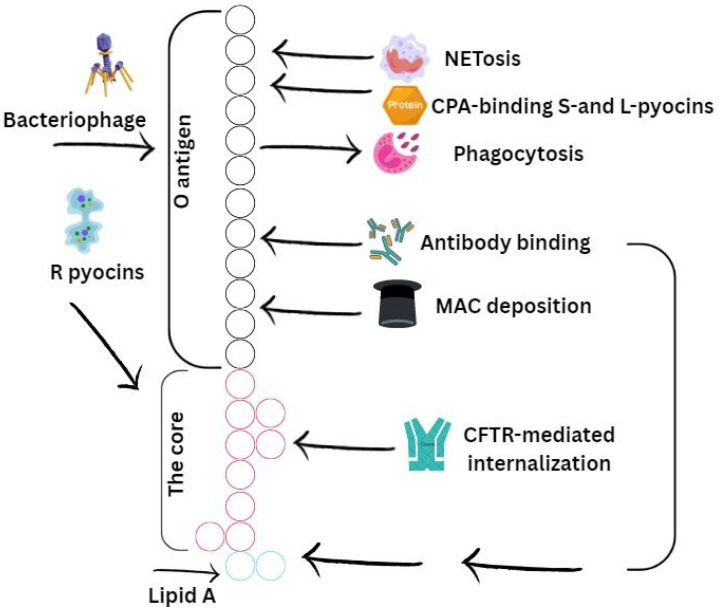
Schematic representation of the O-specific antigen, core (bark) region, and lipid A constituting the LPS of *P. aeruginosa*. Their interaction with host immune mechanisms is presented. Stimulatory or activating effects induced by specific LPS regions are indicated with arrows, while inhibitory effects are represented by T-shaped arrows. The O-specific antigen is illustrated in black, the core region in pink, and lipid A in green. NETs—neutrophil extracellular traps; CPA—common polysaccharide antigen; MAC—membrane attack complex.

Additionally, lipopolysaccharide contributes to the pathogenesis of *P. aeruginosa* by interacting with host receptors, inhibiting host defense mechanisms, and influencing biofilm biogenesis (Figure 2).

### 3.2. Type III Secretion and Invasiveness

**Figure 3:**
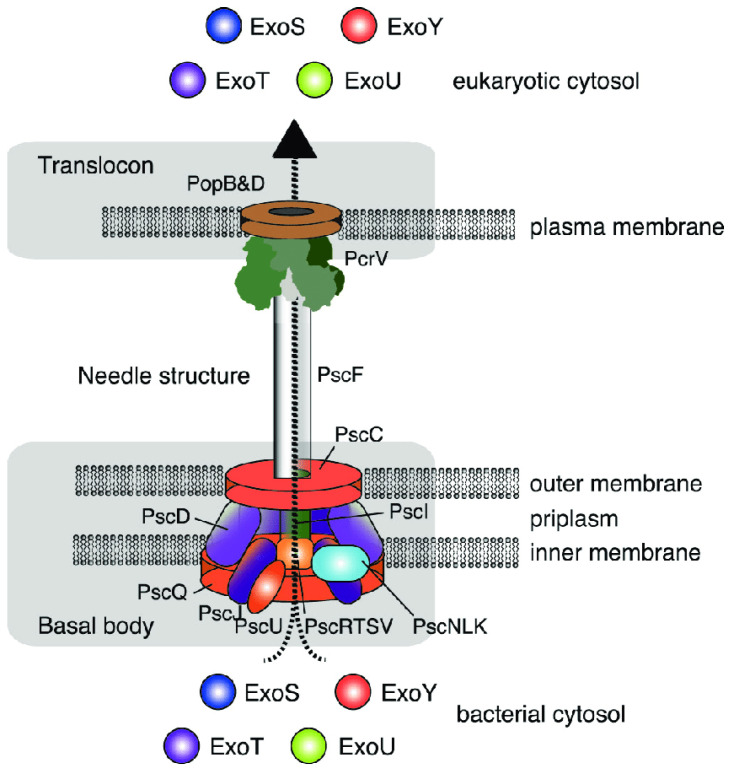
Structure of the type III secretion system in *P. aeruginosa*. Engel and Balachandran [6]. Copyrights, Elsevier.

Among the most important virulence factors of *P. aeruginosa* are effector proteins (exoenzymes, also known as cytotoxins) secreted via the type III secretion system (T3SS). The T3SS is a “hollow molecular needle” that delivers toxins directly into the host cell cytosol. It consists of several proteins forming a macromolecular complex that spans the bacterial membranes, periplasmic space, peptidoglycan layer, and host cell membrane. In addition to the secretion apparatus, the T3SS includes secretory toxins and regulatory components (Figure 3).

Through the type III secretion system, one or more exotoxins are “injected” directly into the eukaryotic cell. Four such effector proteins have been identified in *P. aeruginosa*, namely exoenzyme S (ExoS), exoenzyme T (ExoT), exoenzyme U (ExoU), and exoenzyme Y (ExoY) [6,43].

Although ExoU is produced by only a subset (24–42%) of clinical *P. aeruginosa* isolates [44], it is considered the most significant T3SS cytotoxin due to its major impact on disease severity. ExoU is associated with severe conditions such as acute lung injury, sepsis, and lethality [45]. It possesses phospholipase A2 activity, which irreversibly damages host cell membranes, causing rapid cell death. This cytotoxic effect targets both phagocytes and epithelial cells, aiding in pathogen spread and preventing immune clearance [46]. Additionally, ExoU triggers a proinflammatory response by increasing eicosanoid production in epithelial cells and neutrophils (Neu). It activates the NF-κB pathway, promotes IL-8 secretion during infection, and enhances neutrophil migration into infected lung epithelium [46,47].

ExoT is the most abundant T3SS effector cytotoxin, produced by nearly all clinical *P. aeruginosa* isolates (92–100%) [44]. However, it alone cannot sustain bacterial persistence in the lung [48]. ExoT is a bifunctional exotoxin with both GTPase-activating protein (GAP) and adenosine diphosphate ribosyl transferase (ADPRT) activities, which work together to inhibit phagocytosis and disrupt epithelial barriers [46]. The GAP domain inactivates three small GTPases (Rac, Rho, and Cdc42), which are crucial for maintaining host cell cytoskeleton integrity. This results in reversible disruption of the actin cytoskeleton, inhibiting cell migration and causing cell rounding. Inactivation of Rho also affects cytokinesis. Additionally, ExoT’s GAP activity, in conjunction with ExoS, plays a role in inhibiting effector injection feedback [49]. ExoT specifically ribosylates two adaptor proteins, Crk-I and Crk-II, which are involved in phagocytosis, focal adhesion, and cell migration [50]. Furthermore, ExoT enhances IFN-γ production by NK cells in the lung [51].

ExoS is produced by 58–72% of clinical *P. aeruginosa* isolates [44], and its expression has been linked to chronic infections and worse clinical outcomes in cystic fibrosis (CF) patients [52]. Like ExoT, ExoS is bifunctional, with both GTPase-activating protein (GAP) and adenosine diphosphate ribosyl transferase (ADPRT) activities [46]. The GAP activity of ExoS targets the same three GTPases as ExoT, but its ADPRT domain has a broader range of cellular targets, causing diverse harmful effects on host cells, including cell death, disruption of the actin cytoskeleton, inhibition of DNA synthesis, and interference with vesicle trafficking and endocytosis [46]. Early in infection, ExoS is primarily injected into neutrophils, where its ADPRT activity is a key factor in preventing phagocytosis [53]. As the infection progresses, ExoS is injected into type I pneumocytes, leading to a breakdown of the pulmonary-vascular barrier [53]. ExoS also ribosylates and inactivates the ERM family of proteins, which are involved in motility, phagocytosis, adhesion, and maintaining cell shape [46]. Finally, ExoS activates the TLR2 and TLR4 signaling pathways [54]. ExoS and ExoT are dual-function enzymes with both GTPase and ribosyltransferase. Their ribosyltransferase activity leads to inhibition of host cell DNA synthesis, changes in its actin cytoskeleton, and apoptosis [39,40,41].

ExoY is the second most abundant T3SS effector cytotoxin, expressed by over 89% of *P. aeruginosa* clinical isolates [55]. It functions as a soluble adenylate cyclase that elevates intracellular levels of multiple cyclic nucleotides (cAMP, cCMP, cGMP, and cUMP) upon injection into mammalian cells due to the activation of host protein kinases [56,57,58]. In the lungs, ExoY’s activity results in several pathological effects, including irreversible disassembly of actin microtubules, cell necrosis, disruption of the endothelial barrier, lung function impairment, and terminal dysfunction [55]. Additionally, ExoY inhibits the activation of transforming growth factor β-activated kinase 1 (TAK1), leading to reduced production of proinflammatory cytokines by macrophages and epithelial cells. It also modulates the host immune response by delaying NF-κB and caspase-1 activation [59].

ExoS, ExoT, ExoU, and ExoY destroy the endothelial layer in addition to the epithelial barrier. Destruction of these layers allows the pathogen to spread hematogenously, leading to disseminated infection and ultimately to septic shock [60]. *P. aeruginosa* has been considered an extracellular pathogen [61,62], but recently, strains capable of entering and multiplying in eukaryotic cells (invasive phenotype) have been increasingly identified. The most common invasive strains produce ExoS, whereas non-invasive and cytotoxic strains are most commonly found to have the cytotoxin ExoU, which has the ability to rapidly induce cytotoxicity [62,63].

The question of the relationship between the type III secretion system and eukaryotic cell invasion is unclear. The data are conflicting. Earlier studies suggested a relationship between ExoY, ExoS, and ExoT and the invasiveness of strains. This is questioned by the results of other authors. For example, ExoS has been suggested by some studies to be associated with internalization and has the ability to facilitate invasion [64]. However, other authors describe ExoS as an anti-internalization factor [65], also associated with antiphagocytic activity [66]. ExoT has been reported in the literature to inhibit cytotoxicity [64] or alternatively, internalization and phagocytosis [65,66,67]. It is possible that conflicting data in the literature are related to the bifunctionality of this cytotoxin. Data on the relationship between the type III secretory system and invasiveness are based on only a few model strains examined (RAO1, RAC, RA103, RA14) in combination with several eukaryotic cell models (human epithelial cells, rabbit epithelial cells, MDCK cells, HeLa cells, and macrophages). RAO1 has been shown to produce three of the four effector molecules, ExoU, ExoS, and ExoT, but not ExoU. Despite the ability to synthesize these exotoxins, this strain is invasive [68]. Strain RA103 is of interest, as there are conflicting literature data on it. Some authors classify it as an invasive strain and others as a non-invasive strain [65,67]. It has been shown to have two of the four effector proteins, ExoS and ExoT [65]. The RAC strain is known to be invasive. Strain RA14 produces ExoU but remains extracellular and cytotoxic [64]. The relationship between bacterial regulatory mechanisms and invasiveness is not yet fully established. For example, ExoA regulates the secretion of ExoS and ExoT [66]. When the synthesis of these proteins is intense, bacteria may exhibit a cytotoxic phenotype, and when suppressed, bacteria may develop an invasive phenotype. Also, intercellular bacterial signaling can alter the invasiveness of a given P. aeruginosa strain [66,69]. Besides the effect of gene regulation, there are likely many other factors. For example, no explanation has been found for why at the second hour after infection with a wild-type *P. aeruginosa* strain synthesizing ExoY and with its isogenic ExoY-deficient mutant, bacterial invasion is inhibited, but at the fourth hour after infection, despite disruption of the eukaryotic cell actin, invasion is recorded [66].

The essential role of type III secretory system effector proteins in interactions with the eukaryotic cell has emerged, but the processes are not yet fully understood. It is fundamentally possible that the same mechanisms of actin cytoskeleton disruption by ExoY, ExoS, and ExoT, in some cases in combination with other factors, lead to inhibition of phagocytosis as a protective reaction against phagocytes. In other circumstances, for example, when the expression of these genes is repressed, invasiveness is stimulated, enabling the occupation of new niches in host cells.

In summary, Table 1 presents the role of the aforementioned virulence factors in the pathogenesis of *P. aeruginosa*.

## 4. Adaptations of *P. aeruginosa* to Chronic Lung Infection of CF Patients

The prolonged presence of *P. aeruginosa* in the respiratory system correlates with complex bacterial adaptation strategies, such as biofilm formation, resistance or tolerance to antibacterial agents, and increased mutation frequency. The virulence factors of the bacterium depend on the stage of the infection process. These adaptations are the result of selection during the inflammatory process in the lungs and subsequent antibiotic treatment. During the transition from the acute to the chronic phase of infection, *P. aeruginosa* modifies its survival strategies [70].

In cystic fibrosis, the algG gene in the alginate biosynthetic operon, which is required for the incorporation of L-glucuronate residues into alginate, undergoes changes in amino acid sequences that are part of the domain responsible for carbohydrate binding and sugar degradation. The master regulators for quorum sensing, lasR and rhlR, also frequently show genetic changes in people with chronic infections [71]. Decreased motility, decreased exotoxin A production, changes in the lipopolysaccharide O antigen, and modifications in lipid A are common changes in *P. aeruginosa* strains from long-term lung infection in CF patients [72]. Reduced virulence of *P. aeruginosa* affects the metabolic processes of the bacterium. In isolates with mutations derived from CF patients, an increase in transcription of genes or proteins related to fatty acid and amino acid metabolism as well as energy production was detected. Of particular interest were increases in the expression of genes related to the anaerobic arginine deiminase pathway, anaerobic oxidation (e.g., OprF protein for nitrate, azurin, and cytochrome c551 peroxidase), microaerobic oxidation (e.g., cytochrome cbb3 oxidase), and the tricarboxylic and glyoxylate cycles. A link between increased transcription of the anaerobic regulatory gene anr and activation of ANR-regulated genes was found. There appeared to be an adaptation to stable gene expression required for bacterial growth in the lungs of CF patients [3].

### 4.1. Hypermutability

The genetic diversity of pathogens in the lungs of CF patients is thought to be related specifically to the phenomenon of hypermutability. Here, due to imperfections in DNA repair or error prevention mechanisms, microorganisms have a significantly increased level of spontaneous mutations [73]. There is evidence from both in vitro and in vivo studies that these mutator phenotypes can confer evolutionary advantages when bacteria encounter novel environments or adverse situations. Such mutator cells have been found to occur in bacterial colonies of *P. aeruginosa* at a frequency of 105 due to random changes in their DNA. The increase in antimicrobial resistance in these mutator strains from patients with CF and other chronic infections has been particularly well studied [74,75]. Chronic infections with *P. aeruginosa* pose treatment difficulties. Up to 60% of isolates from CF patients have a hypermutator phenotype. The cause is often related to defects in genes such as mutS, mutL, and mutD. Although these hypermutator isolates are less virulent in in vivo settings, patients have difficulty with lung function and curative therapies are often unsuccessful [76].

These hypermutations lead to greater genetic diversification in the bacterial population. In addition, strains defective in DNA repair have a greater potential to become mucoid, amplifying their virulence in chronic infections, especially in CF patients. Mutations can occur in multiple genes, but selective pressure for adaptation in the CF lung follows, and in later isolates of *P. aeruginosa* from CF patients, some of the mutations are found significantly more frequently [76]. In the course of chronic infections, two of the genes regularly subject to mutations are mucA and lasR. Functional defects in these genes lead to the development of a mucoid phenotype and the inability of the bacterium to respond to changes in its population density, known as quorum-sensing. These changes are important for the persistence of P. aeruginosa in the chronic phase of infection [71,77]. The pelA gene responsible for an enzyme that plays a key role in biofilm formation and dispersal contains the most single nucleotide changes of all 19 genes. Alterations in the pelA protein are widespread in the *P. aeruginosa* population [71]. Mutational processes cause a number of phenotypic changes in CF isolates. These include increased alginate formation and the development of mucoid forms, impaired quorum sensing, absence of motility, lack of type III secretion system effector proteins, alterations in the O-antigenic structure of lipopolysaccharide, low levels of aggressiveness, and limited potential for in vitro biofilm formation and antibiotic resistance [75,77]. Hypermutations also aid bacterial adaptation to the complex environment of the lung, allowing them to survive under different conditions. There are many mutations in key genes that support the transition from acute to chronic virulence. This intense mutability often results in the loss of some virulence functions that are characteristic of acute *P. aeruginosa* infection [78]. Selected hypermutants that are strictly specialized to the lung niche in CF patients have a reduced capacity to spread to new niches; the hypermutability of *P. aeruginosa* may amplify the ability of the bacterium to colonize under conditions of selective pressure characteristic of chronic cystic fibrosis airway infection. This, in turn, may lead to lung deterioration in CF patients in the prolonged presence of the infection [79].

### 4.2. Diversification of Phenotypes

**Figure 4:**
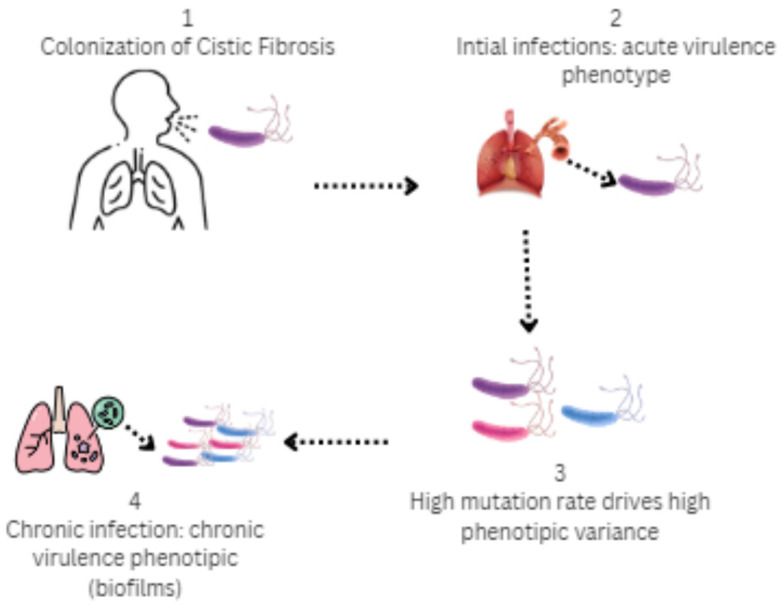
Stages of colonization by *P. aeruginosa* in the airways in cystic fibrosis: 1 (Colonization)—*P. aeruginosa* exhibits significant genetic and phenotypic adaptations that enable it to form complex and diverse bacterial populations; 2 (Initial infection)—Virulence phenotype (type III secretion, flagellum, motile); 3 (High phenotypic variance)—Selective pressures of CF airways over time, antibiotic treatment and nutrient content; 4 (Chronic infection)—Mucoid, immotile phenotype, biofilm formation, and type VI secretions.

Hypermutability creates a rich basis for the selection of diverse phenotypes in the conditions of different habitats in the cystic fibrosis lung. Diverse ecological conditions (such as sites with access to air) stimulate the selection of strains with different degrees of adaptation in terms of virulence and metabolic functions [3]. In prolonged respiratory infections with repeated exacerbations and new colonizations, bacterial populations change their characteristics over time. Strains isolated at the onset of disease often differ markedly from those found in subsequent stages when patients’ health is compromised [80]. Adaptations of *P. aeruginosa*, both at the genetic and phenotypic level, lead to the formation of complex and diverse bacterial populations that are extremely difficult to treat (Figure 4) [78,81]. Comparison of early isolates of *P. aeruginosa* with their clonal descendants from the same patient 90 months later revealed changes indicating a reduced ability to induce acute infections related to the expression of genes responsible for type III secretion, bacterial communication, and mobility [82]. In parallel with reduced aggressiveness, expression of mucoid genes increases and resistance to antimicrobials increases [83]. The mucoid phenotype represents one of the *P. aeruginosa* variants characteristic of CF. The genetic basis behind this phenotype has been well studied, highlighting the key role of mutations in the mucA gene [77]. The mucoid forms are thought to enhance resistance to long-term colonization. In some strains found in critically ill patients, there is an absence of motility organelles. This absence of flagella or pili probably results from mutations in the rpoN gene or in the genes responsible for their formation. Such strains are resistant to phagocytosis [70,84].

Long-term respiratory tract infections in CF patients caused by *P. aeruginosa* often show delayed bacterial growth. This delay is thought to improve patient functionality and enhance bacterial resistance. There are different evolutionary pathways and mechanisms associated with changes in growth rate that are caused by transcriptional level changes and mutations, depending on the stage of strain adaptation [85]. For example, growth acceleration makes bacteria more sensitive to antibiotics. Adaptive evolution dramatically alters growth characteristics and cellular activity. Most of the adapted clinical isolates have retarded growth, which is also associated with enhanced biofilm formation and antibiotic resistance, indicating the importance of this phenotype in chronic infections [86]. In some *P. aeruginosa* strains from patients with CF or other chronic bronchial infections, diversity in colony type has been found. Some of the colonies are mucinous, others have a rough surface, and others are pale. The formation of small colony variants by a given strain alongside larger, mucoid colonies is an adaptive phenomenon associated with some chronic infections, including those with *Pseudomonas* and *Staphylococcus* spp. in CF. Such strains are usually associated with impaired lung function and are often characterized by increased resistance to antimicrobials, in particular aminoglycosides. Bacteria of a given strain localized in small colonies have multiple pili, leading to increased motility, efficient adhesion, and biofilm formation [87,88]. The ability of *P. aeruginosa* to form subpopulations of small colonies likely plays a key role in its strategy for penetration and persistence in diverse environments. However, little is known to date about the functional role of the phenotype of these colonies, which factors drive it, and its potential importance for adaptation. The phenotype of these colonies helps to rebalance redox processes, suggesting its involvement in adaptive mechanisms [89].

Studies of the metabolic spectrum of clinical isolates of *P. aeruginosa* reveal rich heterogeneity in metabolic pathways, highlighting the role of spatial and chemical diversity in the environment during the evolutionary process in CF lungs. This metabolic diversity is consistent with expanded phenotypic and genotypic heterogeneity. It reaches its peak in the initial stages of infection when adaptive changes reshape bacterial physiology to respond to conditions in CF [86,90]. In the respiratory system, *P. aeruginosa* undergoes significant metabolic adaptation due to the nutritional and stress characteristics of the lungs [86]. Comparative study of the proteomes and transcriptomes of isogenic mutator and non-mutator clones has shown that the adaptation response of *P. aeruginosa* in the lungs is characterized by significant metabolic changes. These changes likely contribute to the development of variants with optimized metabolic activity under the conditions of limited access to oxygen that are typical of lungs in patients with advanced cystic fibrosis [46].

Genes related to metabolism show increased expression levels. When the transcriptome of isolates derived from early stages in the course of infection is compared with isolates from the same patient that represent later stages, additional changes that occurred during persistence of the bacterium in the lungs in CF are revealed. When comparing early isolates with later isolates, approximately 15% of *P. aeruginosa* genes (814 gene transcripts; 293 upregulated and 521 downregulated) were found to show expression differences. Many of the genes related to metabolic processes (such as energy metabolism and amino acid and fatty acid metabolism) as well as transport systems show upregulation [91]. This is likely a reflection of selective pressures in a given environment. In advanced phase mutant clones, expression levels of proteins related to metabolic processes are increased. This includes proteins and/or enzymes of the arginine deiminase (ADI) pathway, energy metabolism, amino acid processing, fatty acids, tricarboxylic acid cycle intermediates, energy activity, and transport mechanisms [46]. This metabolic profile likely corresponds to oxygen-limited conditions in the lungs of CF patients. In addition, *P. aeruginosa* strains isolated from the respiratory system of CF patients show increased susceptibility to oxidative stress from the external environment, yet they possess increased resistance to antibiotics under conditions of insufficient oxygen. Another set of characteristics that are adaptive for successful colonization and persistence in CF are independent of the presence or absence of oxygen in the environment [92]. Forms of *P. aeruginosa* that exhibit auxotrophy are more commonly observed in prolonged infections. Auxotrophic organisms need certain amino acids or vitamins to grow. Some strains of *P. aeruginosa* isolated from CF patients have also been observed to develop auxotrophy, and these bacteria need certain amino acids—such as methionine, lysine and arginine—for their growth. It is thought that such auxotrophic forms may arise because of the high concentrations of amino acids in the lungs of patients, leading to the breakdown of the selective pressure for synthesis of these amino acids by the bacterium itself [93].

### 4.3. Persistence

In patients with CF, chronic respiratory tract infection caused by *P. aeruginosa* usually cannot be controlled by clinical administration of appropriate antimicrobial therapy, although pathogenic strains are often sensitive to the antibiotics tested in vitro. This is related to the phenomenon of persistence. Bacterial populations may contain cells that neither grow nor die in the presence of bactericidal agents and thus exhibit tolerance to many drugs. These cells are called persister cells. Persister cells have the phenotype of resting cells, not mutants, and are found in all microbial pathogens. They represent a small fraction of the population but are the only cells that can survive treatment with high concentrations of bactericidal drugs [94,95]. Previous studies have shown the role of persisters in antibiotic tolerance [50]. Such cells also play an essential role in biofilm-associated infections. Studies on different bacterial species have shown that the amount of persisters increases with culture density and can reach about 1% in the stationary phase or in biofilms of *P. aeruginosa*, of *E. coli*, and of *S. aureus* [14]. The majority of patients with long-standing respiratory infections have been found to have elevated levels of persistent cells, and they are also more tolerant to antimicrobial drugs [96]. In bacteria isolated from a patient 96 months after the first isolation of *P. aeruginosa*, an increase in the number of these cells was observed in the exponential phase, the stationary phase, and the biofilm [97]. The structure of biofilms limits the penetration of antibiotics. This, as well as the presence of persister cells, determines the resistance of biofilms against the action of antibiotics [98]. In prolonged infections, the latest *P. aeruginosa* strains isolated from a patient often show increased presence of drug-resistant persister cells [99]. This initially conditioned the assumption that these strains were more resistant in nature. However, observations have shown that they are not more resistant to antibiotics when compared using classical tests. The key to their resistance appears to be their ability to form such cells [94]. Numerous phenotypic and genetic processes contribute to the formation of persister cells. Their formation can increase the resistance of a particular strain, resulting in unsuccessful treatment of respiratory disease in CF [100]. In persisters, there is a decrease in the activity of certain proteins and gene transcripts that are responsible for characteristics that determine the aggressiveness of the bacterium. This includes the type III secretion system (T3SS) and the phenazine and pyochelin synthesis machinery, as well as elastase (with reduced activity), rhamnosyltransferase (with reduced rhlA activity), and flagellar components (with reduced FliC and FliD levels) [101]. The long-term presence of *P. aeruginosa* in the airways of CF patients often results in serious lung damage, which can be fatal. In the setting of prolonged infection, the environment is likely to harbor variants of *P. aeruginosa* that are better adapted to the inflamed tissues of the respiratory system. Proteome and transcriptome studies of initial aggressive strains of *P. aeruginosa* compared to the less virulent mutants of later stages would help us to understand adaptive changes in the gene activity of this bacterium [46].

## 5. Treatment with Antimicrobial Drugs of CF Patients with Bronchopulmonary Infections Caused by *P. aeruginosa*

Inhaled antibiotics have been used to treat chronic respiratory tract infections since the 1940s. The earliest experience with them involved the use of aerosol antibiotics designed for parenteral administration [102,103]. In patients with CF, *P. aeruginosa* often develops resistance if treated with a single antibiotic (such as tobramycin, ceftazidime, or ciprofloxacin) and the concurrent use of two or more drugs from different classes of antibiotics is recommended [104]. Tsifansky (2008) developed different microparticles containing different combinations of antibiotics in different amounts. Such combinations are good for treating several types of microorganisms that cannot be eradicated by a single antibiotic. *P. aeruginosa* is a leading etiologic agent of a number of acute and chronic respiratory infections with therapy problems. This is because of its innate resistance to many groups of antimicrobial drugs, as well as its ability to easily acquire resistance to other antibiotics [105,106]. In CF patients, the resistance levels of *P. aeruginosa* to ceftazidime, cefapime, amikacin, and gentamicin were 19–21%, 18–20%, 12–13%, and 18–20%, respectively. Resistance to carbapenems had the highest rate (32–35%) for the period from 2013. According to the literature data, resistance levels to third- and fourth-generation cephalosporins, amikacin, and gentamicin decreased significantly compared to an increased resistance to carbapenems [107]. The main cause of resistance to commonly used drugs (penicillins, monobactams, third-generation antibiotics, and fourth-generation cephalosporins) is the presence of AmpC β-lactamases [106]. Recent evidence suggests that the use of aerosol antibiotics is the most effective treatment for bronchopulmonary infections [108,109].

### 5.1. Aerosol Antibiotics in CF

**Figure 5:**
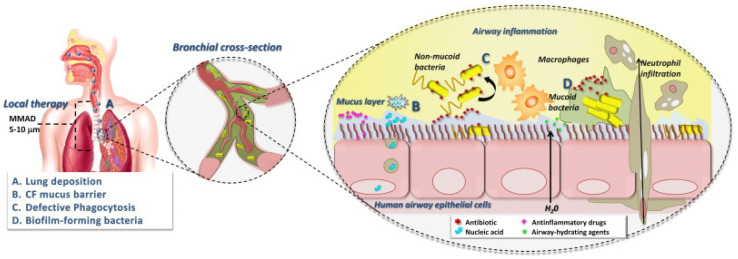
Factors determining the success of inhaled antimicrobial therapy in CF. d’Angelo [113]. Copyrights, American Psychological Association.

Antibiotics in aerosol form facilitate the delivery of extremely high doses directly into infected respiratory organs. The use of inhaled antibiotics in CF patients is advantageous. Aerosol administration of drugs has been preferred in recent years for these patients [110]. Inhaled medications reach the site of infection directly in high concentrations with minimal systemic exposure and toxicity, overcoming some resistance mechanisms [111]. The success of inhaled therapy in CF patients depends on the site and amount of drug in the airways, penetration of drugs through the mucus covering the airways, and interaction with cells and avoidance of macrophages [20,112]. The bacterial biofilm is also important for drug penetration (Figure 5) [113].

Prolonged administration of inhaled antibiotics has been shown to significantly increase forced expiratory volume in 1 s (FEO1), as well as duration and quality of life in CF. It is still unclear which form of antibiotics, powdered or nebulized, is more appropriate for treatment [113]. Currently, several good inhaled preparations are used in medicine (Table 2), mainly tobramycin and colistin [10]. The tobramycin inhalation solution is used in patients with cystic fibrosis who suffer from long-term *P. aeruginosa* infection and are older than 6 years [114]. Regardless of the established indications for administration, there is a possibility of resistance development. For example, a relative trend towards an increase in the frequency of resistant isolates has been observed after administration of the preparation in three consecutive cycles. In the analysis of MPCs for strains detected before and after the start of tobramycin therapy, the results were equivocal. Resistance to this antibiotic can be caused by different mechanisms, such as limited access to the bacteria, more efficient clearance of the substance from bacterial cells, genetic changes, etc. [16].

According to Taccetti (2021), despite significant advances in the use of inhaled antibiotics such as colistin, tobramycin, aztreonam lysine, and levofloxacin, pulmonary infections are still a leading cause of death in cystic fibrosis patients. The use of these antibiotics as maintenance therapy for chronic *P. aeruginosa* infections allows high drug concentration directly in the lungs, improving pharmacokinetic and pharmacodynamic parameters and reducing toxicity. The frequent use of inhaled antibiotics for the prevention of pulmonary exacerbations is also gaining popularity. This review discusses the efficacy and safety of available inhaled antibiotics, with particular attention to strategies for eradication of *P. aeruginosa* and other pathogens, as well as the effects of long-term therapy on chronic infections and prevention of pulmonary exacerbations [115].

Cystic fibrosis is characterized by chronic infections and inflammation of the airways that lead to progressive deterioration of lung function. Although the life expectancy of patients has significantly increased in recent decades, most still die from respiratory failure. The primary goal of antibiotic therapy is to preserve and, if possible, improve lung function through daily management of pulmonary disease and prompt, intensive treatment of exacerbations [110]. The introduction of new therapies targeting the defective CFTR protein has significantly changed the management of cystic fibrosis, but antibiotic therapy remains a major component of the treatment of pulmonary infections. The use of inhaled antibiotics allows high concentrations to accumulate in lung tissue with minimal systemic toxicity. However, the limited choices of inhaled antibiotics and the gradual decline in their effectiveness over time create a need for the development of new antibacterial agents [115,116]. The study by Taccetti (2021) highlights the significant advantages of inhaled antibiotic therapy over systemic (oral or intravenous) therapy. The main advantages include achieving high concentrations of active substance in the lungs, reducing bacterial density, and improving patients’ clinical symptoms and quality of life. Despite the proven efficacy of antibiotics such as tobramycin and aztreonam, their use in a cyclical regimen (28 days of intake followed by rest) results in temporary declines in lung function. There is a growing trend towards the use of continuous inhaled antibiotic therapy or combination regimens with alternating different antibiotics, showing improved clinical outcomes. The need for eradication of *P. aeruginosa* in the early stages of infection to prevent chronic colonization should also be emphasized. Early antibiotic therapy has been shown to significantly increase the chances of successful pathogen elimination and improve long-term clinical outcomes [117]. However, approximately 10–40% of patients fail to clear the infection after initial treatment. Taccetti (2021) examined different antibiotic treatment strategies, including combination regimens with oral and inhaled antibiotics. In addition to *P. aeruginosa*, other pathogens such as *Staphylococcus aureus* and *Burkholderia cepacia* complex can also lead to severe pulmonary complications, necessitating the development of targeted therapeutic protocols [115]. This study highlights the role of inhaled antibiotics in preventing pulmonary exacerbations. Despite the widespread use of tobramycin and aztreonam, patients continue to suffer recurrent exacerbations, pointing to the need for new and more effective therapeutic approaches. Levofloxacin, as the newest approved inhaled antibiotic, has shown promising results in reducing the incidence of exacerbations and improving lung function.

Another important area of research is the interaction between antibiotic therapy and the pulmonary microbiota. The mucosal environment and the microbial community in the lungs have a significant impact on the effectiveness of antibiotics. Studies have shown that inhaled antibiotics can alter the microbiome of the lungs, and the presence of certain bacterial species can affect therapeutic response. There are still no clear guidelines on how these factors should be taken into account when designing personalized therapeutic regimens [115]. The study findings highlight the importance of inhaled antibiotic therapy in improving long-term clinical outcomes in patients with cystic fibrosis. Although new therapies targeting CFTR mutations are transforming disease management, antibiotic therapy remains a critical component of the overall therapeutic approach. Future development of new inhaled antibiotics and improved strategies for the management of pulmonary infections are expected, including a better understanding of the microbiological and immunological mechanisms influencing the effectiveness of antibiotic therapy [118]. According to Karakonstantis and Saridakis (2020), colistin is one of the few remaining effective treatments against carbapenem-resistant *Acinetobacter baumannii*, but the emergence of heteroresistance raises serious concerns about the effectiveness of therapy. In a systematic review and meta-analysis, they examined the incidence, mechanisms, and therapeutic implications of colistin heteroresistance in *Acinetobacter* spp. [119]. Data from 15 studies were analyzed, and the mean incidence of heteroresistance was found to be 33%, but it varied considerably between studies. Previous exposure to colistin was associated with a higher incidence of resistant subpopulations. Heteroresistance is primarily due to chromosomal mutations in the pmrAB and lpx genes that result in modification or loss of lipopolysaccharide, as well as increased expression of efflux pumps. In laboratory settings, no colistin dosing regimen has been able to prevent the emergence of resistant subpopulations, but in clinical settings, resistance from heteroresistant isolates during treatment is less frequently observed. Studies linking heteroresistance to clinical and microbiological outcomes in patients are still lacking [119]. The data suggest that heteroresistance is inherent in *Acinetobacter* spp., as it is found even in isolates that have not been exposed to colistin. However, the frequency of this phenomenon remains variable in different studies, probably due to differences in detection methods as well as the specificity of the strains studied. Although resistant subpopulations can occur during treatment, in most cases, these mutations result in significant biological costs to the bacteria, which explains why clinical colistin resistance still remains relatively low. Colistin-based combination therapies (e.g., with rifampicin, carbapenems, or ampicillin/sulbactam) have shown promising results in the laboratory, preventing the development of resistant subpopulations. However, clinical trials have so far shown no significant improvement in therapeutic outcomes. One possible reason for this is that colistin-resistant bacterial subpopulations may not be clinically relevant in most cases due to their reduced virulence [119]. The findings of this study highlight the need for further studies to assess the clinical significance of heteroresistance, the incidence of colistin-resistant strains during treatment, and possible associations with clinical outcomes. The introduction of standardized detection methods, such as mini-PAP, may facilitate the identification of heteroresistant strains in routine practice.

### 5.2. Long-Term Effects of P. aeruginosa Eradication in CF

The long-term effects of *P. aeruginosa* eradication in patients with cystic fibrosis are of key importance for the prognosis of the disease and the development of pulmonary complications. Although early elimination of the pathogen is considered an important therapeutic goal, a growing number of studies have shown that removal of the infection alone does not always result in complete recovery of lung function and resolution of the inflammatory process. Residual neutrophil activity and ongoing inflammation even after successful eradication can lead to long-term structural damage and increased risk of reinfection [7]. The underlying mechanism behind this process is the continued presence of activated neutrophils in the airways, even after removal of *P. aeruginosa*. Neutrophils secrete a large amount of proteolytic enzymes, such as neutrophil elastase, which degrade lung tissue and lead to the progression of bronchiectasis. The inflammatory response remains active, meaning that tissue damage continues even when the bacterium is no longer detectable in bronchoalveolar lavage fluid. Studies have shown that high neutrophil elastase levels after eradication are associated with increased risk of future infections and worsening of structural lung changes [120]. Even when infection is successfully eliminated, the pulmonary environment remains favorable for recolonization with *P. aeruginosa* or other pathogenic microorganisms. Inflammatory mediators and epithelial destruction weaken the local immune response, facilitating reinfection. In addition, ongoing inflammation can alter the composition of the mucosal layer, reducing the ability of the lungs to clear pathogens and maintain a sterile environment [121]. This means that even after the administration of intensive antibiotic therapy, patients remain at increased risk of recurrence, highlighting the need for long-term follow-up and additional interventions.

Another important aspect is that inflammatory activity and structural changes in the lungs after eradication may continue to worsen regardless of the presence of infection. Studies have shown that children with cystic fibrosis who had high neutrophil elastase levels after eradication developed more severe bronchiectasis within the following year [122]. This suggests that standard eradication strategies may not be sufficient for long-term preservation of lung function and that combination treatment including both infection control and limitation of inflammation is necessary. Therapeutic approaches to address this problem may include the use of anti-inflammatory agents, such as macrolides or neutrophil protease inhibitors, which may reduce tissue damage. It is also possible to implement additional strategies, such as mucoregulatory therapies or antioxidants, which may improve the pulmonary environment and reduce the propensity for recurrent infections. Existing studies on CFTR modulators also suggest that these drugs may have a positive effect on inflammation and reduce the incidence of *P. aeruginosa* infections, but their long-term effect on neutrophil activity is not yet fully understood [123]. Continued inflammation after *P. aeruginosa* eradication is a serious problem that may compromise the long-term success of therapy in patients with cystic fibrosis. Understanding the complex interactions between inflammation, infection, and structural changes in the lungs is essential for developing more effective therapeutic strategies that not only eliminate infection but also protect the lungs from progressive damage [123]. A study by Garratt (2021) analyzed the impact of *P. aeruginosa* eradication on airway inflammation in children with cystic fibrosis and evaluated the relationship between persistent neutrophil elastase activity and deterioration of lung structures. All participants underwent a standard regimen of infection eradication and subsequent bronchoalveolar lavage evaluation to track changes in inflammatory response and structural changes in the lungs [123]. The results of the study show that eradication was successful in 84.1% of cases, with median neutrophil elastase activity decreasing significantly after treatment. However, neutrophil elastase levels remained elevated in some patients, which was associated with a greater risk of *P. aeruginosa* reinfection within one year. Furthermore, persistent activity of this enzyme and the initial bronchiectasis index on computed tomography were the best predictors of progression of bronchiectasis regardless of successful eradication. These results underscore the fact that elimination of *P. aeruginosa* is not always sufficient to achieve long-term stability of lung function, as the inflammatory process may persist after removal of the pathogen. Inflammatory mediators, including neutrophil elastase, remain active and can cause progressive destruction of lung tissue, leading to irreversible damage in the long term. These observations suggest the need for personalized therapeutic approaches that not only focus on eliminating infection but also on controlling chronic inflammation. Potential interventions include the use of anti-inflammatory agents, neutrophil protease inhibitors, or adjuvants such as nitric oxide, which may have both anti-inflammatory and antibacterial effects. The development of such strategies may help reduce long-term disability and improve prognosis in patients with cystic fibrosis [7].

The need for personalized therapy in these patients is of particular importance as standard antimicrobial regimens do not always prevent disease progression. Even with successful elimination of *P. aeruginosa*, the presence of persistent inflammation sets the stage for further damage to the lung structure and predisposes patients to new infections. This highlights the need for an integrated approach that combines antimicrobial therapy with control of the inflammatory process to prevent chronic lung damage and improve patients’ quality of life.

## 6. Conclusions

The management of pulmonary infections in cystic fibrosis remains a complex clinical challenge, despite recent therapeutic advances targeting CFTR dysfunction. Inhaled antibiotics have demonstrated notable clinical benefits, including improved lung function, reduced bacterial load, and enhanced quality of life. However, the persistence of airway inflammation following apparent microbial eradication suggests that infection control alone is insufficient to prevent long-term pulmonary decline. Residual neutrophilic inflammation, particularly sustained neutrophil elastase activity, contributes to continued tissue damage and the progression of structural abnormalities such as bronchiectasis, even in the absence of detectable pathogens. These findings underscore the necessity of therapeutic strategies that extend beyond microbial eradication to address the underlying inflammatory milieu and its long-term consequences.

Furthermore, the emergence of antimicrobial resistance—including heteroresistant subpopulations—highlights the urgent need for novel antimicrobial agents and optimized treatment regimens. Personalized approaches that integrate anti-inflammatory therapies, mucoregulatory agents, and possibly CFTR modulators may offer improved outcomes by simultaneously targeting infection, inflammation, and epithelial dysfunction.

A comprehensive understanding of the interplay between microbial dynamics, host immune responses, and airway remodeling is essential for the development of future therapeutic strategies aimed at preserving lung function and improving long-term prognosis in CF patients.

## Figures and Tables

**Table 1:** Major virulence factors of *P. aeruginosa* and their role in pathogenesis.

Secretion System	Virulence Factor	Host Target	Function/Role in Pathogenesis
Type II	Elastase A (LasA) or staphylolysin	Matrix proteins	Gly-Gly 20 kDa metallopeptidase; enhances the elastinolytic activity of LasB; secreted as proenzyme.
Type II	Elastase B (LasB)	Matrix proteins	33kDa Zn-metalloprotease; degrades elastin and fibronectin.
Type II	Exotoxin A	Elongation factor-2 (cytosol)	ADP-ribosylates elongation factor 2 → blocks protein synthesis; induces apoptosis.
Type II	Phospholipase C (PLC-H)	Cell membranes	Hemolytic and cytotoxic properties; increases vascular permeability and neutrophil activation.
Type II	Protease IV	Complement proteins, IgG	Serine protease; degrades immune molecules; expression is quorum sensing-dependent.
Type II	Pyocyanin	Various cells	Redox-active phenazine; disrupts respiration; calcium homeostasis; induces apoptosis.
Type II	Pyoverdine	Cellular iron	High-affinity siderophore; scavenges Fe^3+^ and regulates toxin expression.
Type III	ExoS	Cytoskeleton	RhoGAP and ADP-ribosyltransferase; inhibits phagocytosis, promotes apoptosis.
Type III	ExoT	Crk proteins	Dual enzymatic activity; inhibits cell internalization and immune signaling.
Type III	ExoU	Cell membrane	Potent phospholipase; causes rapid host cell lysis and inflammation.
Type III	ExoY	Cytoskeleton	Secreted adenyl cyclase; increases concentration of intracellular cAMP in host cells; actin cytoskeleton disruption and increased endothelial permeability.
	Alginate	Biofilm matrix	Mucoid polysaccharide; contributes to chronic infection and antibiotic resistance.
	Flagella	TLR5, Ipaf receptors	Provides motility; triggers innate immune activation (NF-κB, caspase-1).
	Type IV Pili (T4P)	Cell surface	Mediates adhesion and twitching motility; activates virulence gene expression via the Chp chemo-sensory system.

**Table 2:** Inhaled antibiotics for CF. Addition of new ones from post-recent years; DPI—dry powder inhale.

Medication	Form	Trade Name	Dosage	Dosage Interval	Time of Administration
**Aztreonam**	solution	Cayston^®^	75 mg	every 8 h	2–3 min
**Colystine**	solution	Colomycin^®^ Promixin^®^	80 mg (1,000,000 E)	every 8 or 12 h	4–7 min
	DPI	Colobreathe^®^	125 mg (1,662,500 E)	every 12 h	1–2 min
**Tobramycin**	solution	TOBI300^®^ Bramitob^®^ Actitob^®^ Tobrineb^®^	300 mg/5 mL 300 mg/4 mL	every 12 h	Per. 20 min
	DPI	TIPTOBI^®^	112 mg	every 12 h	per. 15 min
